# Hybrid fuzzy clustering and temporal deep learning framework for multi-parameter forecasting in industrial thermal processes

**DOI:** 10.3389/frai.2026.1826705

**Published:** 2026-08-17

**Authors:** V. Vignesh, G. V. Narendran, R. Senthil Kumar, R. Sitharthan

**Affiliations:** 1 School of Electrical Engineering, Vellore Institute of Technology, Chennai, Tamil Nadu, India; 2 Centre for Smart Grid Technologies, Vellore Institute of Technology, Chennai, Tamil Nadu, India

**Keywords:** blast furnace, deep learning, forecasting, Fuzzy C-Means, predictive modelling

## Abstract

Blast furnace (BF) operation involves strongly coupled thermal and chemical processes governed by nonlinear heat transfer, gas–solid reactions, and dynamic operational control. Accurate real-time prediction of key thermal and gas parameters is essential for maintaining furnace stability, improving energy efficiency, and reducing carbon emissions. However, most existing studies focus on single-parameter forecasting and fail to adequately capture the heterogeneous temporal dynamics and strong process coupling inherent in blast furnace operations. In this paper, a hybrid fuzzy clustering and temporal deep learning framework is proposed for multi-parameter forecasting. This framework integrates Fuzzy C-Means (FCM) clustering with temporal deep learning models, such as Nonlinear Autoregressive with Exogenous Inputs (NARX), Recurrent Neural Network (RNN), Long Short-Term Memory (LSTM), and Gated Recurrent Unit (GRU). The dataset consists of 43,396 industrial Distributed Control System (DCS) samples collected from an operating BF. FCM is employed to identify different operating regimes and generate fuzzy membership values, which are subsequently utilized as sample weights during model training. The performance of the proposed models is evaluated using Mean Absolute Error (MAE), Mean Squared Error (MSE), Root Mean Squared Error (RMSE), and coefficient of determination (*R*^2^). Among the developed models, the FCM-GRU model achieved the best overall forecasting performance, attaining *R*^2^ values of 0.602, 0.355, 0.656, and 0.912 for the prediction of Hot Metal Temperature (HMT), silicon content (Si), CO, and CO₂ respectively. The novelty of the proposed work lies in integrating fuzzy membership-based operational-state identification with temporal deep learning architectures for simultaneous forecasting of multiple blast furnace parameters. The obtained results demonstrate the feasibility of the proposed framework for real-time process monitoring and predictive decision support in blast furnace operations.

## 1 Introduction

Steel production is one of the most energy- and carbon-intensive industrial activities. In the traditional blast furnace-basic oxygen furnace process, molten iron (hot metal) is generated by blast furnaces, and later transformed into steel in basic oxygen furnaces. According to the World Steel Association’s Sustainability Indicators 2024, world crude steel output dropped by 4.3% in 2022 relative to 2021, while demand recovery was slow in 2023. At the same time, the energy intensity of the industry rose to 21.27 GJ per tonne of crude steel in 2023, while CO₂ emissions intensity remained stuck at around 1.92 tonnes CO₂ per tonne of crude steel, underlining the industry’s struggle in making significant environmental progress (World Steel Association, 2024).

Process fluctuations and unexpected downtimes in BF represent significant operational problems for the steelmakers. HMT, the percentage of silicon (Si), and alterations in the composition of the top-gas (CO, CO_2_) not only spoil the quality, but it also affects the profits as well. The recent statistics in the industry confirm that unplanned shutdown of a single blast furnace may take USD 700,000 to 1.2 million per hour, depending on the size of the plant and the market conditions (GrowthLenz, 2025). The large scale steel plants waste nearly 27 h a month on unexpected failures, totalling nearly 324 h annually, which results in the more financial losses (Siemens, 2024).

In addition to the financial problems, operational disruptions in BF processes can also lead to increased energy consumption and environmental emissions. The steel production industry is contributing approximately 7–9% of the total CO_2_ emissions in the world, hence it is a key point of attention in solving the problem of climate change (Cypag, 2024). According to experts, inefficient use of the industrial assets all around the world has resulted nearly USD 10 billion losses, with cost increasing by 15% since the previous year (Wood Mackenzie, 2024). The conventional control methods are difficult to manage the complex process fluctuations and the time delays in the BF operations.

This paper investigates the use of FCM as a method of data clustering, and deep learning models, such as NARX, RNN, LSTM, and GRU, to forecast the BF parameters, such as HMT, Si percent, CO, and CO_2_. While implemented in real time environments, the proposed system can minimize operational interruptions, lower operational costs and helps to emission reduction. This study aligns with SDG 9, as it improves the performance of BFs by predicting key findings using DL and FCM. Accurate forecasting of BF parameters, which enables to minimize the process disruptions and enhance the productivity (Liu and Sun, 2025). Beyond that, the proposed strategy supports SDG 13 (climate action), due to more accurate the prediction, that minimize the inefficient operations and reducing emissions. The enhanced monitoring the BF behavior, can reduce the unnecessary coke consumption which is the primary cause of CO2 emissions in steel plants (Alghieth, 2025). Early prediction of rising silicon content or falling HMT enables operators to adjust fuel rate, oxygen enrichment, or burden composition before thermal imbalance propagates through the furnace.

Due to their enhanced capability to handle complex, non-linear, and temporal dynamics, several Artificial Intelligence (AI) methods, including Machine Learning (ML) and DL have been utilized in the recent years for BF state forecasting. Diverse data-driven methods have been developed (Giannetti et al., 2025) to predict chief BF parameters. Guo et al. (2025) presented a multi-variable data-driven predictive model for predicting entire blast furnace status, emphasizing the capability of such models to identify complicated process interactions. As per a survey conducted by Qin et al. (2024), models such as NARX provide interpretability through the use of well-defined inputs variables while DL models such as LSTMs and GRUs are best at learning temporal characteristics.

One of the critical research areas has been Si content and HMT prediction as essential indicators of the BF thermal condition. Raza et al. (2025) and Meng et al. (2024) employed complete ML pipelines and optimized GBDT models, respectively, to accomplish precise Si prediction. Hao et al. (2025) proposed a multiscale fusion convolutional network to learn complex temporal patterns for silicon content. Paixão et al. (2025) developed intra- and inter-cast HMT forecasting using ML. In parallel, gas-related parameter prediction has also become prominent to ensure efficiency and emissions control. Liu et al. (2025) used denoising multiscale spectral graph wavelet neural networks to predict gas utilization ratio. Zhang et al. (2025) utilized a digital twin-based framework for carbon emission concentration forecasting.

Extending the work in these studies, the proposed system addresses the simultaneous forecasting of multiple thermochemical variables. The existing studies such as Liu and Sun (2025) and Hao et al. (2025) have targeted single variables prediction using specialized designs. Although the advanced architectures like multiscale convolutional networks and digital twin frameworks have been explored in literature, such designs are commonly computationally complex and difficult to implement for practical deployment in real-time industrial systems. The main contribution of the proposed work lies in benchmarking simple and efficient network architectures for a multi-output prediction. This establishes a clear performance with a computationally efficient architecture that is acceptable in real-time. The proposed models are trained based on the entire range of operating conditions and enhance accuracy and reliability in predicting furnace status.

Shi Q. et al. (2024) proposed a data-driven methodology to predict BF heat signals, and demonstrated that AI based methods can help to maintain the stable operations. Similarly, there is also a study on applying RNNs and their variations, including LSTM and GRU, for time-series prediction in industrial manufacturing process such as steelmaking, in order to learn the sequential dynamics that play a key role in the BF control (Guo et al., 2025; Qin et al., 2024). Despite the merits of the traditional models such as the neural networks (NN), Support Vectors Machine (SVM), Random Forests (RF) and the Decision Trees (DT) to monitor BF operations, none of the models can completely address all the operational problems especially those arising due to the highly nonlinear and time-delayed characteristics of BFs. Due to it, the interest in hybrid frameworks utilizing the strengths of several ML approaches has been increasing. Meradi (2025) demonstrated an experimental validation of the convolutional neural networks (CNNs) to detect faults in power equipment of BF by integrating thermal imaging and current measurements to enhance accuracy. Based on these studies, even though models such as NN, SVM, RF, DT, logistic regression, *k*-nearest neighbours (KNN), and naive Bayes offer various advantages to BF monitoring, they do not focus on all the operational problems. NN and RF are suitable in large and complex data sets whereas classical regression-oriented methods suitable for smaller data sets. Signal processing methods like wavelet transforms are employed to extract and detect features to train models of NN.

Recent studies have shown that the hybrid DL frameworks can effectively predict complex industrial thermal processes. Sun et al. (2026) proposed a hybrid DL framework for high-precision prediction of transient thermal-flow behavior to enhance the forecasting accuracy Under dynamic operating situations. Zhao et al. (2024) combined the ML and computational fluid dynamics (CFD) to predict real-time heat transfer in coke making processes, demonstrating the advantages of combining a data-driven with a physics-based approach, Qiu et al. (2025) proposed a hybrid DL algorithm for solving the problem of transient heat conduction and enhanced prediction accuracy and robustness under different thermal conditions. Despite these advances, the existing studies primarily focus on single-process thermal characteristics and do not address multi-parameter forecasting under varying industrial operating regimes. To address this issue, a hybrid Fuzzy Clustering and Temporal DL approaches is proposed, which combines the temporal DL models such as NARX, RNN, LSTM, and GRU with FCM clustering to learn different furnace operating regimes and nonlinear temporal relationships.

The hybrid method is embedded to model and forecast important BF parameters like HMT, silicon composition (Si%), and gaseous compositions (CO, CO₂). Predicting silicon (Si) content in hot metal is particularly challenging due to delayed thermochemical reactions, variations in coke reactivity, slag chemistry, and non-uniform burden distribution with in the furnace. Incorporating FCM enables efficient management of process uncertainty and the intrinsic fuzziness of BF working states, grouping data points into significant clusters before input into the sequence models. Instead of picking models at random, DL systems were selected due to their stronger ability to grasp complex temporal patterns along with complex dynamics: NARX due to its feedback loops, including external inputs, RNN serving as the core structure behind looped designs, and LSTM or GRU can handle fading signals over extended sequences. The proposed system provides the consistent and energy efficient operation at blast furnaces by combining FCM and DL models.

**Figure 1:**
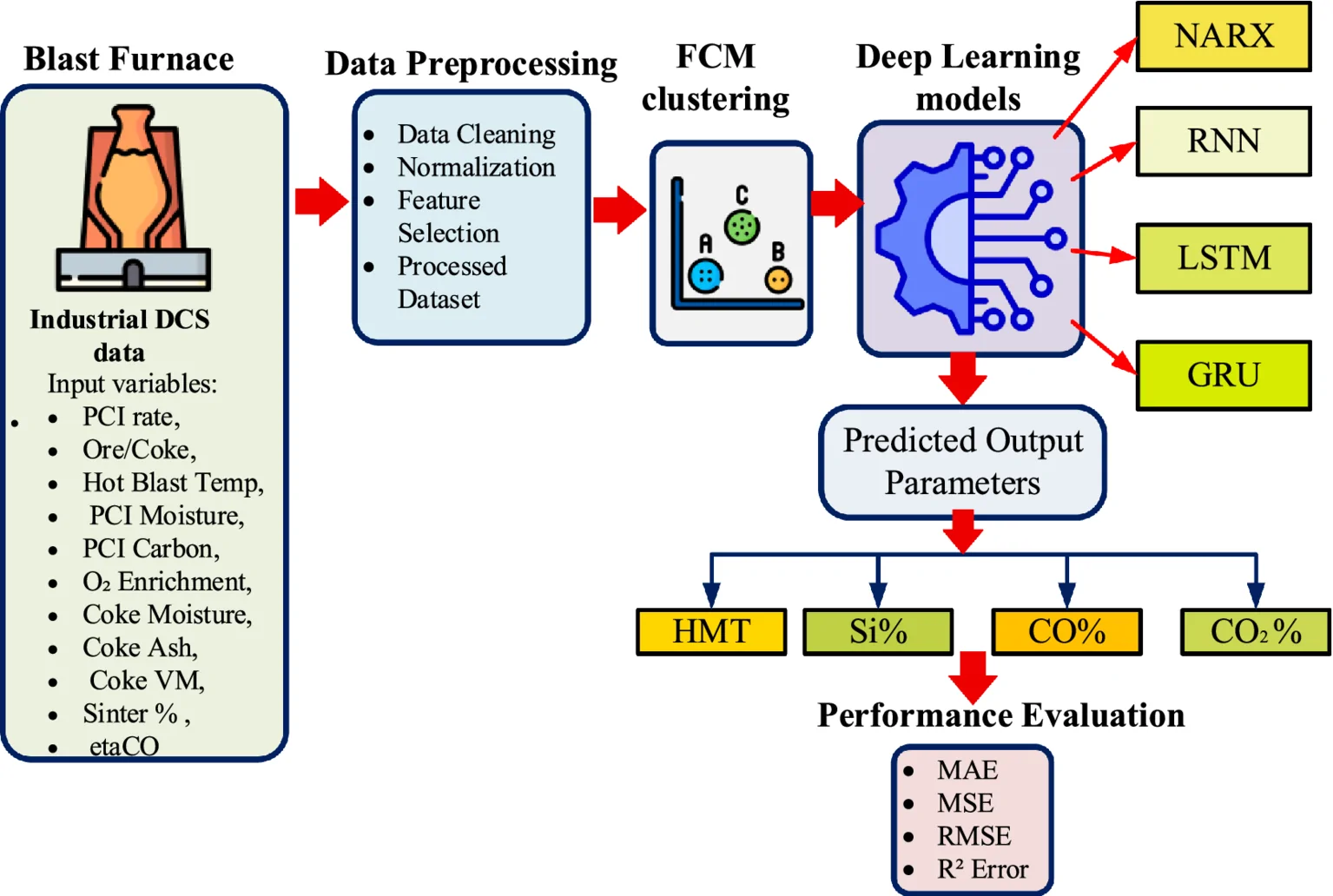
Overall framework of the proposed hybrid FCM–DL system.

Figure 1 demonstrates the Overall framework of the proposed hybrid FCM–DL system to predict the behavior of the blast furnace. The system input is the historic operational data such as PCI rate, ore/coke, hot blast temp, PCI moisture, PCI carbon, O₂ enrichment, coke moisture, coke ash, coke VM, sinter %, and etaCO. These data are initially pre-processed using such operations as data cleaning, normalization, and rescaling to give equal representation to features. Time-lagged features are then built to reflect temporal dependence and dynamic trends that are inherent in the operations of the blast furnaces.

The pre-processed information is then subjected to a FCM in which the data on the operational data are clustered together in regard to their operations into certain clusters depending on the different operating conditions of the furnace. An effective application of such clustering step is to address the uncertainty in process and the vague nature of blast furnace conditions by clustering the similar operating conditions and then train the models (Bhardwaj et al., 2024). These clustered data are then fed to temporal DL models, such as NARX, RNN, LSTM and GRU.

To determine the best performing model, results were evaluated by RMSE, MAE and *R*^2^ scores. Early detection of changes also allows operators to adjust the parameters in advance, as it forces the furnace to operate more efficiently. Traditional statistical models often fail to capture such nonlinear temporal relationships. RNN, LSTM, and GRU are DL models capable of learning the long-term dependency in the time-series data, making them suitable for the modelling the dynamic behavior of BF processes. The proposed FCM-based preprocessing stage identifies distinct furnace operating regimes and incorporates process uncertainty before temporal learning. This enables the DL models to capture heterogeneous operating conditions more effectively than direct model training on unclustered data.

The main contributions of the proposed work are summarized as follows:

- Unlike traditional BF prediction models (Liu and Sun, 2025; Giannetti et al., 2025; Raza et al., 2025; Hao et al., 2025; Paixão et al., 2025), the proposed hybrid FCM-DL framework improves the prediction of key BF parameters under different operating conditions.
- FCM-derived fuzzy membership information increases the feature representation and improves DL model performance compared to existing approaches (Guo et al., 2025; Liu et al., 2025).
- A systematic comparison of NARX, RNN, LSTM, and GRU models identifies the most appropriate architecture for BF parameter forecasting, which has received limited attention in previous studies (Raza et al., 2025; Paixão et al., 2025; Liu et al., 2025).
- The proposed framework is validated using long-duration real-time industrial DCS data, which representing its practical applicability beyond studies based on limited datasets (Raza et al., 2025; Meng et al., 2024; Paixão et al., 2025).
- The developed forecasting framework supports reliable real-time BF monitoring and operational decision-making.

The remainder of this paper is organized as follows: Section 2 is the discussion of the data description and modelling of the BF and the various DL algorithms are used in the prediction of the parameters. The entire flow process of the proposed system is illustrated in section 3. Section 4 addresses the performance metrics of proposed system to find out the accuracy of prediction. Section 5 discusses the effectiveness of the models based on the results that have been obtained. Section 6 presents the comparison of the proposed works with the existing works. Section 7 is the conclusion of the paper, which summarizes the main findings.

## 2 Data description and DL modelling

### 2.1 Data from BF process

The BF ironmaking process is a large scale metallurgical process used to extract molten iron from iron bearing materials like sinter, pellets, lump ore etc. Iron making is a highly dynamic and nonlinear process that is associated with complex thermochemical reactions, gas–solid interactions, and heat transfer processes. During a conventional BF, the raw materials such as iron ore, coke and fluxes are loaded on the top of the blast furnace, and at the bottom, preheated air referred to as hot blast is brought in through tuyeres. The combustion of coke with oxygen in the hot blast generates high temperatures, typically exceeding 2000 °C, which produces reducing gases such as carbon monoxide (CO). These gases rise upward through the furnace and reduce iron oxides present in the burden materials.

This study aims to develop robust data-based model for multi-step predicting of BF parameters. This problem is set up as a supervised, multi-output regression task. Using a historical time-series window of input parameters (like PCI rate and hot blast temperature) and output parameters (Si%, HMT, CO%, CO₂%), the aim is to forecast the future values of the output parameters at the next time step. Mathematically, the forecasting problem can be defined in Equation 1.

$${\hat{y}}_{t}=F(x_{\left(t-1\right)},y_{\left(t-1\right)},\theta )$$ (1)

Where,

${\hat{y}}_{t}$ —predicted vector at time *t*, i.e., [ ${\hat{\mathrm{CO}}}_{t},{\hat{\mathrm{Hmt}}}_{t}\;\mathrm{etc}.]$ , F—hybrid FCM-neural network model, $x_{\left(t-1\right)}$ —vector of exogenous input features at time at *t*−1, $y_{\left(t-1\right)}$ —vector of target values at time *t*−1, incorporated via a one-step lag to create an NARX-style setup, and θ —learned parameters of the model.

HMT is a primary indicator of the thermal condition inside the blast furnace. It is the difference between the amount of heat added to the blast and the amount of heat used or lost in endothermic reactions, slag formation and radiation. The total energy balance may be given in Equation 2.

$$Q_{\mathrm{net}}=Q_{\mathrm{hb}}-(Q_{r}+Q_{s}+Q_{l})$$ (2)

where $Q_{\mathrm{hb}}$ represents the hot blast heat input, $Q_{r}$ denotes the heat consumed by reduction reactions, $Q_{s}$ corresponds to the heat required for slag formation, and $Q_{l}$ is for heat losses to the surroundings. Deviation from the normal operating range of the 1,475–1,485 °C indicating thermal imbalance because of varying hot blast temperature, fuel rate or oxygen enrichment. The resulting $Q_{\mathrm{net}}$ is the thermal condition of the furnace and it is one of the most important parameters which affect the HMT and silicon content in the hot metal. A change in the temperature of hot blast, the rate of fuel injection or in enrichment in the oxygen can greatly change this energy balance and cause changes in the performance of the furnace.

Si Content is also another significant factor indicating silicon transfer efficiency and the thermal conditions in the furnace. Predictions indicating outside the 0.35–0.50% range indicate a shift in the chemical balance of the process, that may influence the fuel rates and operational ratios.

CO_2_ and CO percentages are major gas components used to find the combustion efficiency in BF operations. Suppose that increase in CO% or decrease in CO_2_% indicated that inefficient reduction, which is generally associated with fluctuation of the ore-to-coke ratio or the enrichment of the ore with the oxygen. These reactions are related to Boudouard equilibrium and represented in Equation 3.

$${\mathrm{CO}}_{2+}C⇌2\mathrm{CO}$$ (3)

This reaction shows that, solid carbon is used to reduce carbon dioxide to form carbon monoxide. The reaction is also an important endothermic reaction in the BF, especially in the raceway and lower stack areas, where temperatures are high and carbon monoxide is generated. The CO produced acts as the main reducing gas, that converts iron oxides to metallic iron in the furnace. These thermodynamic relations are not explicitly incorporated in the NN, but it helps to select the input variables and interpret the output of predictions.

The data was taken on the DCS of a running blast furnace in an integrated steel mill in India. Primary characteristics of this dataset and the preprocessing parameters are as in Table 1. The data was a large number of samples of the long-term dynamics of the process to a steady regime. The parameters of key processes have been measured at regular intervals, which provides a closer look at the behavior of the furnace.

**Table 1:** Key dataset and modelling parameters.

Sl. No.	Parameter	Description
1	Dataset size	43,396 samples
2	Input features	PCI rate, ore/coke, hot blast temp, PCI moisture, PCI carbon, O₂ enrichment, coke moisture, coke ash, coke VM, sinter %, etaCO
3	Target variables	Si content, HMT, CO%, CO₂%
4	Sampling interval	10 min
5	Data imputation	Median imputation for missing values
6	Feature engineering	One-step lag features added
7	Scaling method	Robust scaling
8	Clustering technique	FCM with 3 clusters
9	Train/test split	80% training, 20% testing
10	Noise handling	IQR-based outlier removal and moving-average smoothing.

To capture the variability of process in real world conditions, the data has a wide set of operational setpoints of important inputs, such as pulverized coal injection rates and oxygen enrichment levels. Sensor noise has also been considered during analysis to ensure that models would be robust under actual plant conditions. After that, the dataset has been separated for model development, allocating most of it for training while keeping a separate set for testing. They standardized all input features to ensure the stability and effectiveness of model. The HMT, Si, CO, and CO₂ are forecasted based on variations input parameters such as PCI rate, coke rate, HMT, top pressure of BF.

### 2.2 DL models for BF parameter prediction

ML provides a robust method to model and predict blast furnace’s complex, time-sensitive behaviors. There are also several dependent variables, such as the temperature of hot metal, the level of silicon, coke rate, and the top gas composition, which influence the processes of BF. These variables may fluctuate rapidly with the fluctuations in the raw materials, operational requirements, and the limitations on the equipment. Controlling such nonlinearities and delays are usually complex in more conventional process control techniques, whereas the ML-based models tend to excel in identifying temporal patterns and predicting the behavior of a system. This enhances operational stability, energy performance, and quality of products. The proposed method is organized into various primary stages: clustering, model design, fuzzy weighted training, and evaluation.

#### 2.2.1 Fuzzy C-Means (FCM) clustering

Using FCM clustering, the BF dataset has been intensely monitoring the different operational regimes before choosing the NN models. By varying the degrees of membership, this method allows each data point to belong to several clusters, which captures uncertainty and overlap among the different states of furnace operation (Bhardwaj et al., 2024).

The membership degree *u_ik_* of a sample *x_i_* in cluster *k* is expressed in Equation 4.

$$u_{\mathrm{ik}}=\frac{1}{\sum_{j=1}^{c}{\left(\frac{x_{i}-c_{k}}{x_{i}-c_{j}}\right)}^{\frac{2}{m-1}}}$$ (4)

The cluster centers are updated using Equation 5

$$c_{k}=\frac{\sum_{i=1}^{N}u_{\mathrm{ik}}^{m}x_{i}}{\sum_{i=1}^{N}u_{\mathrm{ik}}^{m}}$$ (5)

Where, *m*—fuzziness parameter, *N*—quantity of samples, and *c*—number of clusters.

FCM came up with three operational states that included stable, transitional, and unstable. In the process of training the NN, the membership values were used to give weight to the data points, and this enables each model to provide more emphasis to the data points that are in close relation to its cluster. This approach helps to reduce the forecasting errors in case of sudden changes of processes, and enhancing the robustness of the model (Bhardwaj et al., 2024).

#### 2.2.2 Nonlinear Autoregressive State and Exogenous Inputs (NARX)

The NARX model is an excellent predictor model used in laying out a forecasting model. It is a model that seeks to predict the variables of furnace output, including temperature of the hot metal by means of both their historic values (the autoregressive) and historic values of the external factors (the exogenous), in this case, the blast temperature and coke rate. The NARX is expressed as in Equation 6.

Y(t)=f(y(t−1),y(t−n),u(t−1),u(t−m)) (6)

Where, Y(t) is the predicted variable, and *u*(*t*) is the external input features. This is an excellent arrangement in terms of complex industrial processes, including BFs, in which several interrelated factors influence the output. The NARX model enhances the prediction accuracy by modelling the dynamic relationships between the internal and external variables. This is employed in order to improve on proactive control action and help in optimization of the overall functioning of the furnace (Hamidi and Fakhroleslam, 2025).

#### 2.2.3 Recurrent Neural Network (RNN)

RNNs learn the short-term time-varying connections within the furnace data such as blast pressure, top gas composition, and Hot Metal Temperature point (Waqas and Humphries, 2024). The difference between RNNs and feedforward NN lies in having recurrence networks, where the knowledge of a given state is manipulated by the current state. The update of the hidden state is presented in Equations 7, 8.

$$h_{t}=f(w_{\mathrm{xh}}x_{t}+w_{\mathrm{hh}}h_{t-1}+b_{h})$$ (7)

$$\text{The output is computed as:}y_{t}=W_{\mathrm{hy}}\;h_{t}+b_{y}$$ (8)

where $h_{t}$ is the hidden state at time *t*, $x_{t}$ is the input vector, and *f* is an activation function.

RNNs established the foundation for sequence modelling, enabling the detection of rapid variations in the blast furnace operation. They can be useful in detecting immediate deviations since they are effective in capturing the short variations, e.g., when the gas composition is suddenly changed or that the temperature of the hot metal is fluctuating. However, they tend to struggle with modelling long-term dependencies due to the vanishing gradient problem.

#### 2.2.4 Long Short-Term Memory (LSTM)

The LSTM networks are used to address the vanishing gradient problem (Ławryńczuk and Zarzycki, 2025). These networks are an exclusive form of RNN that applies gating mechanisms. These gates assist in regulating the flow of information, and hence LSTMs have proven to be successful in learning long-term relations in furnace data (Raza et al., 2025). The LSTM Equations are represented in Equations 9–12.

Input gate:

$$i_{t}=\sigma (W_{i}[x_{t},h_{t-1}]+b_{i})$$ (9)

Forget gate:

$$i_{t}=\sigma (W_{f}[x_{t},h_{t-1}]+b_{f})$$ (10)

Cell state update:

$$c_{t}=f_{t}*C_{t-1}+i_{t}*\tanh(W_{c}[x_{t},h_{t-1}]+b_{c})$$ (11)

Output gate:

$$h_{t}=o_{t}*\tanh(C_{t})$$ (12)

Here, $i_{t},f_{t,\;}o_{t}$ regulate the amount of information that is inputted, stored or retrieved in memory or output.

The LSTM networks are also effective when it comes to predicting the behavior of the furnaces, as they identify the long-term trends such as the gradual changes in the coke consumption and the quality of hot metals. They have input, forget, and output gates to control information that is added, stored, or shared at every level. This long-term dependency capturing ability comes into play crucially when we are looking at variables such as the influence of the quality of coke or a gradual increase in silicon content. This is especially when using LSTMs to predict the variables of a furnace, where the temporal dependencies and delayed process impacts are critical.

#### 2.2.5 Gated Recurrent Unit (GRU)

**Figure 2:**
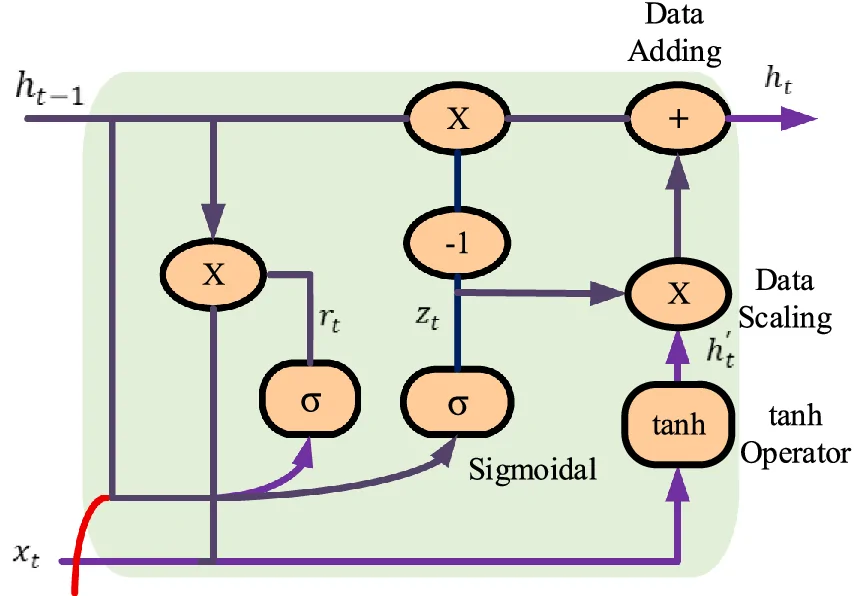
GRU architecture.

GRU is specially developed to capture temporal dependence without the heavy burden of computation. The architecture of the GRU is shown in Figure 2. It simplifies the architecture of the LSTM, merging the input and forget gates into update gate, and the hidden state with the cell state (Balakumar et al., 2026). This design minimizes the number of parameters, which minimizes the amount of computation required, and projects with great accuracy. The expressions of GRU network is mentioned in Equations 13, 14.

$$z_{t=}\sigma (W_{z}[x_{t},h_{t-1}])$$ (13)

$$h_{t=}(1-z_{t})*h_{t-1}+z_{t}*\tanh(W_{h}[x_{t,}r_{t}*h_{t-1}])$$ (14)

Here, σ is sigmoid activation function (outputs values between 0 and 1). $W_{z}$ weight matrix for the update gate and $h_{t-1}$ is previous hidden state.

By achieving the balance between efficiency and accuracy, GRUs are ideal for real-time monitoring and control tasks where quick computations are crucial. Here in blast furnace operations, they can deliver timely short-term predictions without the high training times and hefty memory demands that come with LSTMs. Thus, it is an excellent option for ensuring operational stability through prompt feedback and control.

### 2.3 Deep learning architecture and hyperparameter configuration

Table 2 summarizes the architectural configurations and training hyperparameters employed for the FCM-assisted deep learning models developed for blast furnace parameter forecasting. To ensure a fair comparison, all models were trained using identical optimization settings, including a learning rate of 0.001, batch size of 32, and 50 training epochs.

**Table 2:** Deep learning architecture parameters.

Model	Network structure	Hidden units	Batch size	Epochs	Learning rate	Optimizer	Activation function
FCM–NARX	NARX + dense	32	32	50	0.001	Adam	tanh
FCM–RNN	Simple RNN + dense	64	32	50	0.001	Adam	tanh
FCM–LSTM	LSTM + dense	64	32	50	0.001	Adam	tanh
FCM–GRU	GRU + dense	64	32	50	0.001	Adam	tanh

The Adam optimizer was selected because of its adaptive learning capability and efficient convergence characteristics for nonlinear industrial process data. The tanh activation function was utilized in the recurrent layers to effectively capture temporal dependencies and dynamic process behavior. A Dense output layer with four neurons was used in each model to simultaneously predict Si, HMT, CO%, and CO₂%. FCM clustering was integrated with all models through soft membership weighting, enabling the learning process to account for different blast furnace operating regimes. The hidden unit configuration was selected based on preliminary experiments to achieve a balance between predictive performance and computational complexity.

### 2.4 Hybrid FCM-based framework

FCM is first employed to identify distinct furnace operating regimes and generate fuzzy membership values that represent the degree of association of each data sample with different clusters. These membership values are subsequently used as sample weights during model training, enabling the forecasting models to account for process variability and nonlinear operating conditions. This integration allows the models to better capture complex temporal relationships among blast furnace variables and improves forecasting performance.

The number of FCM clusters was set to *C* = 3, representing different operating states of the blast furnace. The clustering process was performed using soft membership assignment, allowing each sample to belong to multiple clusters with varying degrees of membership. The fuzzification coefficient was set to *m* = 2.0, providing a balance between hard and soft clustering behavior. The maximum number of iterations was fixed at 300, and the convergence tolerance was set to 1 × 10^−5^. The iterative optimization process terminated when the change in cluster centers between successive iterations became smaller than the predefined tolerance or when the maximum iteration limit was reached.

## 3 Modelling procedures

**Figure 3:**
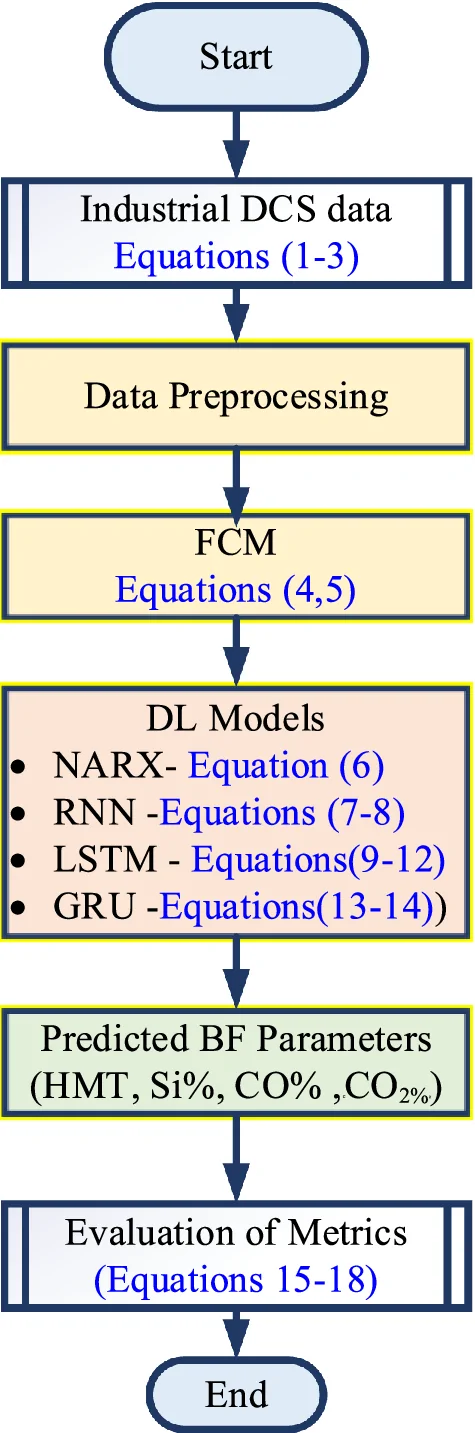
Flow process of the proposed system.

The overall work flow of the proposed hybrid forecasting framework is shown in Figure 3. Initially, the historical BF operational data are acquired from the industrial DCS as represented in Equations 1–3. The collected data are then preprocessed through data cleaning, normalization, and feature extraction before being clustered using FCM algorithm, where the fuzzy membership values and cluster centers are computed using Equations 4–5. The clustered data are subsequently provided as inputs to four DL models such as NARX, RNN, LSTM, and GRU, whose mathematical formulations are represented in Equations 6–14. The developed models perform one-step-ahead forecasting, corresponding to a 10-min prediction horizon to estimate the key BF parameters (HMT, Si%, CO%, and CO₂%). Finally, the forecasting performance is evaluated using MAE, MSE, RMSE, and *R*^2^, as defined in Equations 15–18.

### 3.1 Data acquisition

First, the historical operational data of the DCS of the blast furnace and associated process sensors were collected. This data covered the time-series data of key parameters such as hot blast temperature, wind pressure, oxygen enrichment rates and top gas composition. The inputs plays as a dominant role in predicting the target variables such as HMT, silicon content (Si%), CO%, and CO₂ %.

### 3.2 Data preprocessing and feature engineering

After collecting the raw data, it is then pre-processed to achieve quality and stability of the model. It is performed by filling in the gaps in the values by interpolation, removing blatant outliers and standardizing the data to a normal distribution. The sensor data of a blast furnace is inherently noisy as a result of errors in measurements, disturbances in the process and sensor drift. In order to enhance the reliability of the model, a number of preprocessing steps had been taken. The missing values were processed with Median imputation. The Interquartile Range (IQR) was used to identify outliers and eliminated them. Further, short-term fluctuations, as well as sensor noise were removed by applying a moving average filter. This step is essential to avoid the cases when the features with higher numerical values preponderate over the rest of the features during model training. Time-lagged features have been added to assist the models learn the natural dynamic relationships in the process of the blast furnace.

### 3.3 Model training

In this paper, four neural network architectures, including NARX, LSTM, GRU, and RNN are developed and trained. The pre-processed data set is divided into training data set and testing data set in which training data set contains 80% data and testing data set contains 20% data. Each of the models was trained to relate the parameters of the input process with the desired output variables based on finding multifaceted, non-linear patterns in the historical data.

### 3.4 Multi-output forecasting

Models used are selected after being trained and predict. They considered the most recent sequential input data to generate concurrent estimates of four outputs variables. This method of multi-step prediction provides an overall representation of the internal state of the blast furnace.

### 3.5 Model evaluation

The models were evaluated by comparing the predicted values with actual, untestable test data. Time-series plot has been used to visualize the actual versus predicted values. The analysis of the residual errors (the differences between the expected and the actual events) has been done in detail to assess bias, variance, and overall reliability.

When testing with different operating conditions of the BF, the process variables were gathered to capture realistic variations. Table 3 highlights the benchmarking ranges of critical parameters (that are used as reference levels to gauge the performance). These limits determine the limits of operation of testing models. Any unfamiliar furnace behavior, stable operations, and optimization of the processes could be achieved through monitoring of the changes in parameters such as silicon content, HMT, CO/CO₂ composition, and hot blast conditions.

**Table 3:** Benchmarking ranges of different parameters of dataset.

Sl. No	Parameter	Unit	Min value	Max value	Typical range	Remarks
1	etaCO	—	43.44	48.61	44.5–46.5	Gas utilization efficiency
2	PCI rate	kg/thm	171.42	209.14	180–205	Raceway heat intensity
3	Coke rate	kg/thm	318.36	458.38	340–360	Coke consumption rate
4	Ore/coke	—	4.92	5.76	5.4–5.7	Burden permeability control
5	Hot blast temp	°C	1131.95	1197.55	1,150–1,180	Furnace heat balance
6	O_2_ enrichment	%	8.66	9.68	9.0–9.4	Combustion enhancement
7	CO%	%	23.34	29.35	26.5–28.5	Top gas CO content
8	CO₂%	%	19.08	24.82	22.0–23.5	Top gas CO₂ content
9	Si content	%	0.30	0.70	0.35–0.50	Hot metal silicon content
10	HMT	°C	1,465	1,493	1,475–1,485	Hot Metal Temperature

## 4 Evaluation of models

The statistical performance metrics are used to evaluate the hybrid blast furnace model. The critical parameters are predicted with high accuracy using DL model. The predictive framework is reliable for real-world furnace operation by the performance of regression metrics like RMSE, MAE, MSE, and *R*^2^ error.

### 4.1 Mean Absolute Error (MAE)

The MAE gives the average absolute differences between predicted and actual values. Unlike MSE, MAE treats all errors equally, which makes it less affected by outliers (Serefoglu Cabuk et al., 2024). The MAE is expressed in Equation 15.

$$\mathrm{MAE}=\frac{1}{N}\sum_{i=1}^{N}|y_{i}-{\hat{y}}_{i}|$$ (15)

Where $y_{i}$ —actual value, ${\hat{y}}_{i}$ —predicted value. MAE is robust to extreme values and gives a clear indication of model performance.

### 4.2 Mean Squared Error (MSE)

MSE quantifies the distance between the forecasts and the real ones, thus averaging the squares of the differences. This squaring implies that the contribution of larger errors to the overall score is more significant and MSE is particularly sensitive to outliers (Thieu, 2024). The MSE is expressed in Equation 16.

$$\;\mathrm{MSE}=\frac{1}{N}\sum_{i=1}^{N}{\left(y_{i}-{\hat{y}}_{i}\right)}^{2}$$ (16)

and *N* is the total number of observations.

### 4.3 Root Mean Squared Error (RMSE)

RMSE is simply the square root of the MSE and represented in Equation 17. The RMSE puts the error back into the same units as the values being predicted, which makes the average error easier to understand.

$$\text{RMSE}=\sqrt{\frac{1}{N}\sum_{i=1}^{N}{\left(y_{i}-{\hat{y}}_{i}\right)}^{2}}$$ (17)

It provides a more interpretable sense of average prediction error.

### 4.4 Coefficient of determination (*R*^2^)

The *R*^2^ error indicates the proportion of variance in a dependent variable, which can be predicted by independent variables in a regression model. A value closer to 1 shows the better fit, indicating that the model accounts for significant portion of the variability in the data. The *R*^2^ error is expressed in Equation 18.

$$R^{2}=1-\frac{\sum_{i=1}^{N}{\left(y_{i}-{\hat{y}}_{i}\right)}^{2}}{\sum_{i=1}^{N}{\left(y_{i}-{\bar{y}}_{i}\right)}^{2}}$$ (18)

Here, $\bar{y}$ is the mean of actual values.

## 5 Results and discussion

**Figure 4:**
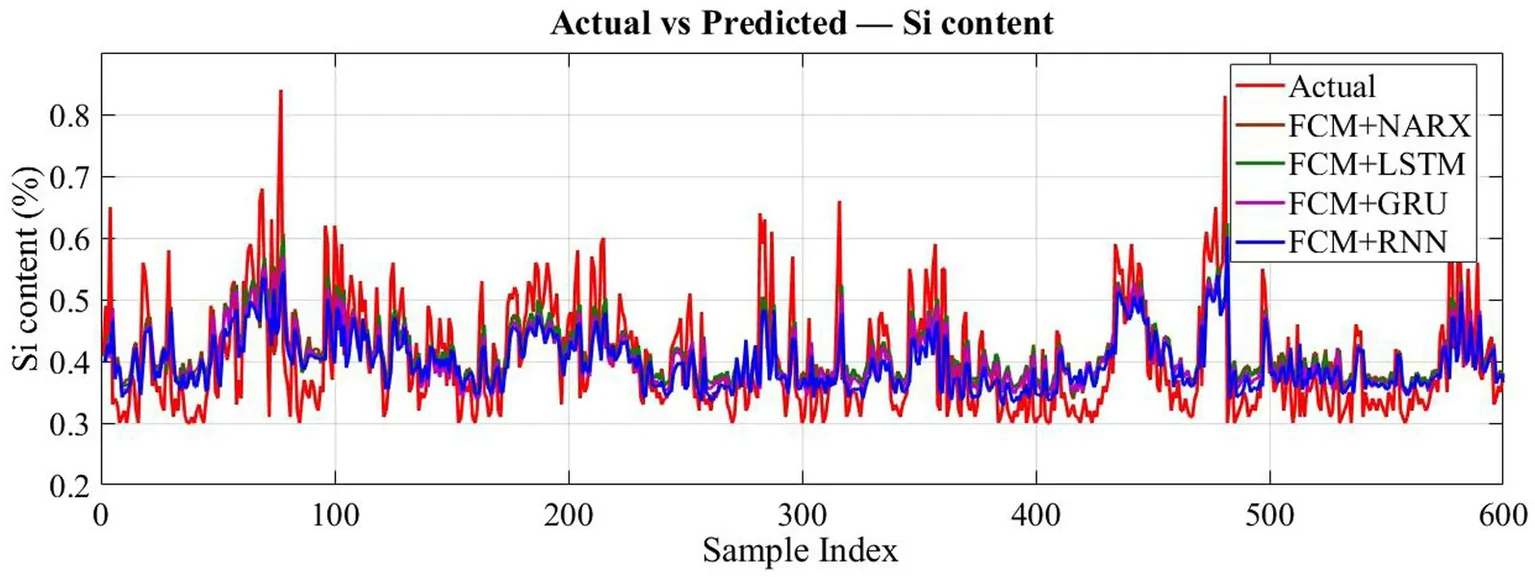
Actual vs. predicted of silicon content.

**Figure 5:**
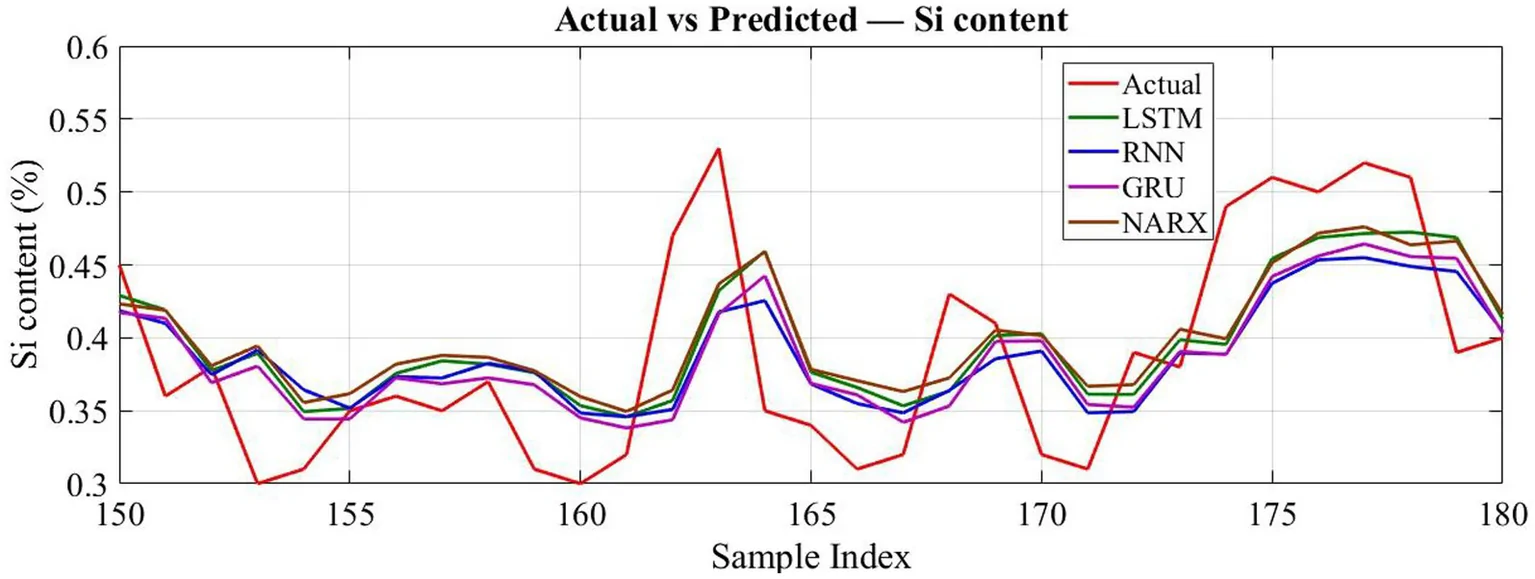
Zoom view of actual vs. predicted of silicon content.

**Figure 6:**
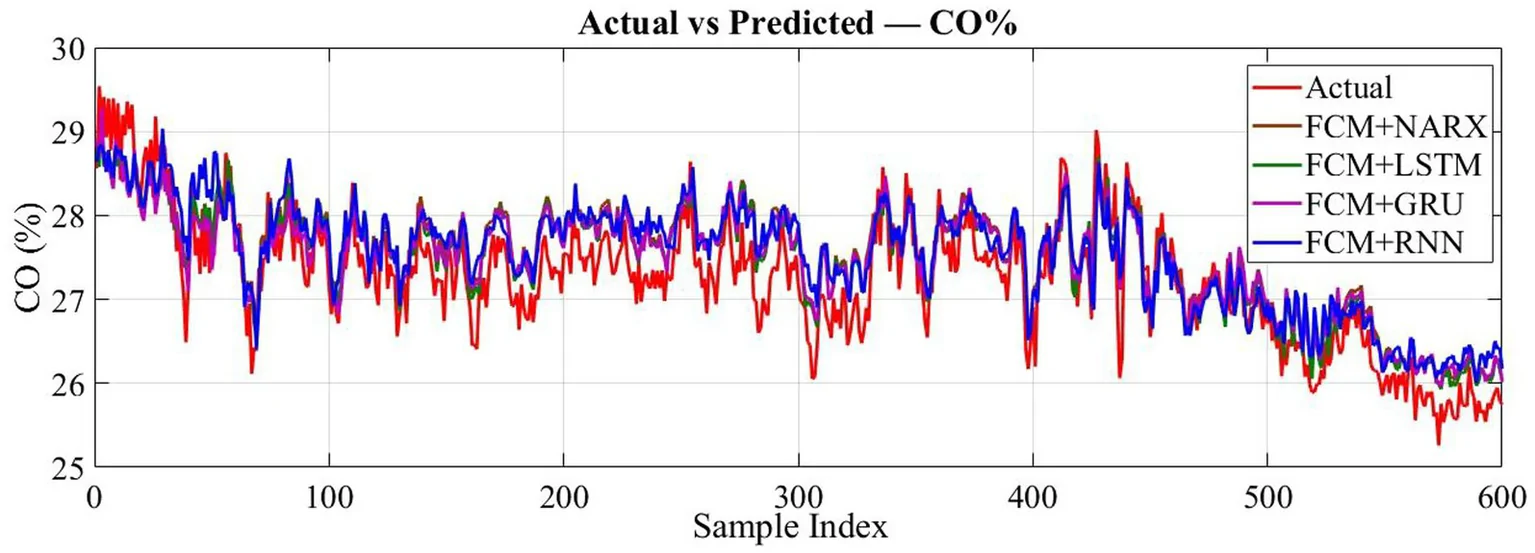
Actual vs. predicted of CO %.

**Figure 7:**
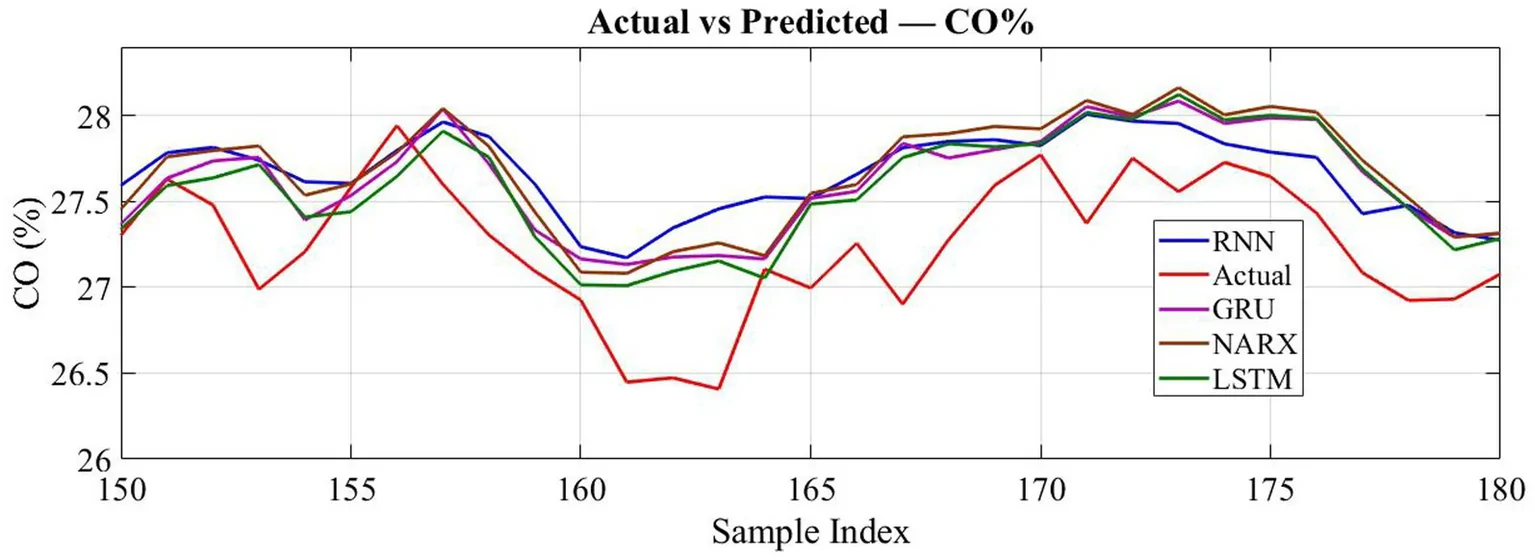
Zoom view of actual vs. predicted of CO %.

This section analyses the predictive ability of the developed models and explains the implication of the findings. Figures 4–7 show the accurate prediction of CO% and Si content of the four models by plotting their forecasted values against the actual data. The GRU, NARX, and LSTM models deliver impressively accurate predictions. Their forecast lines closely follow the actual data, effectively capturing the high-frequency fluctuations and sharp peaks of these process variables. Whereas the RNN model struggled, predicting data that were too smooth and lagging. They could not train on the dynamic range of the data, especially for the volatile Si content, where crucial peaks and troughs are not forecasted accurately.

**Figure 8:**
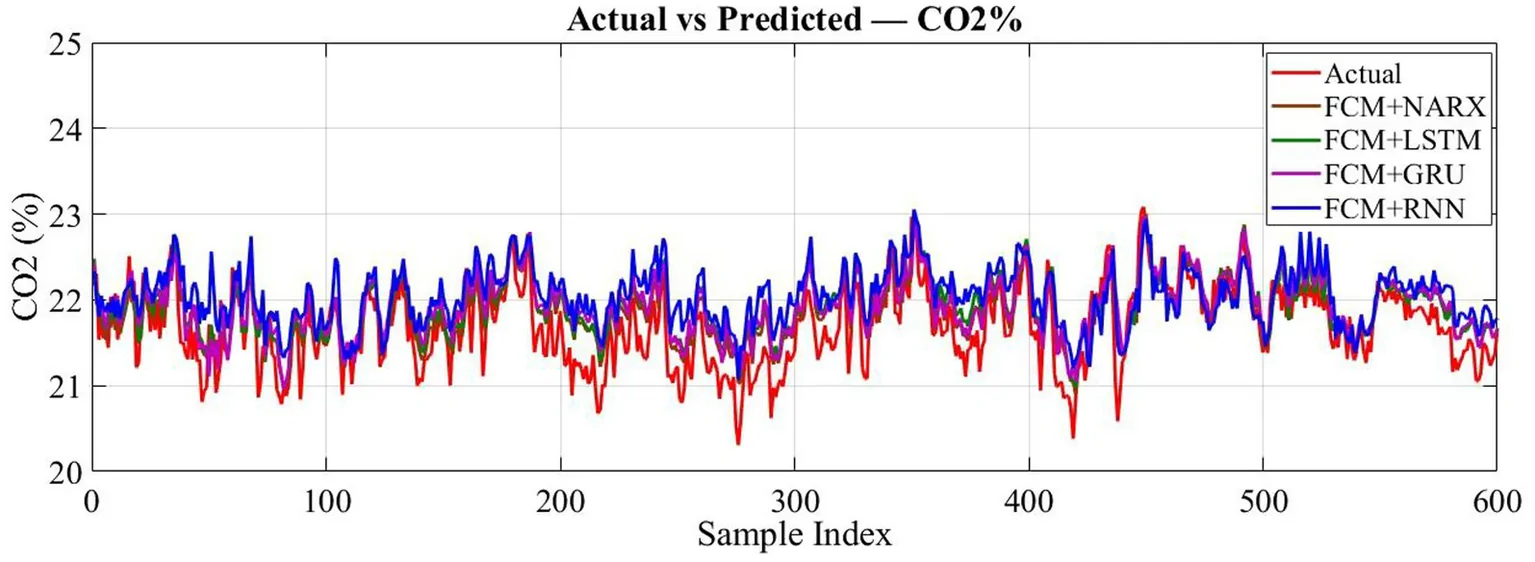
Actual vs. predicted of CO_2_ %.

**Figure 9:**
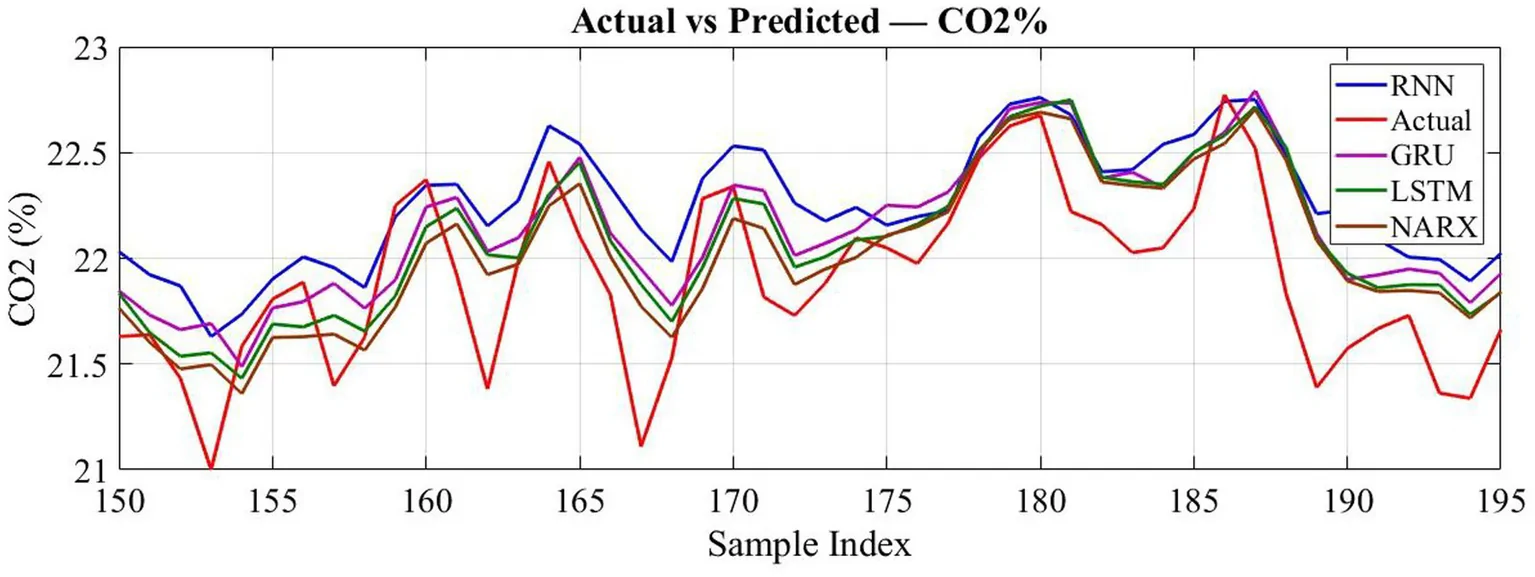
Zoom view of actual vs. predicted of CO_2_ %.

**Figure 10:**
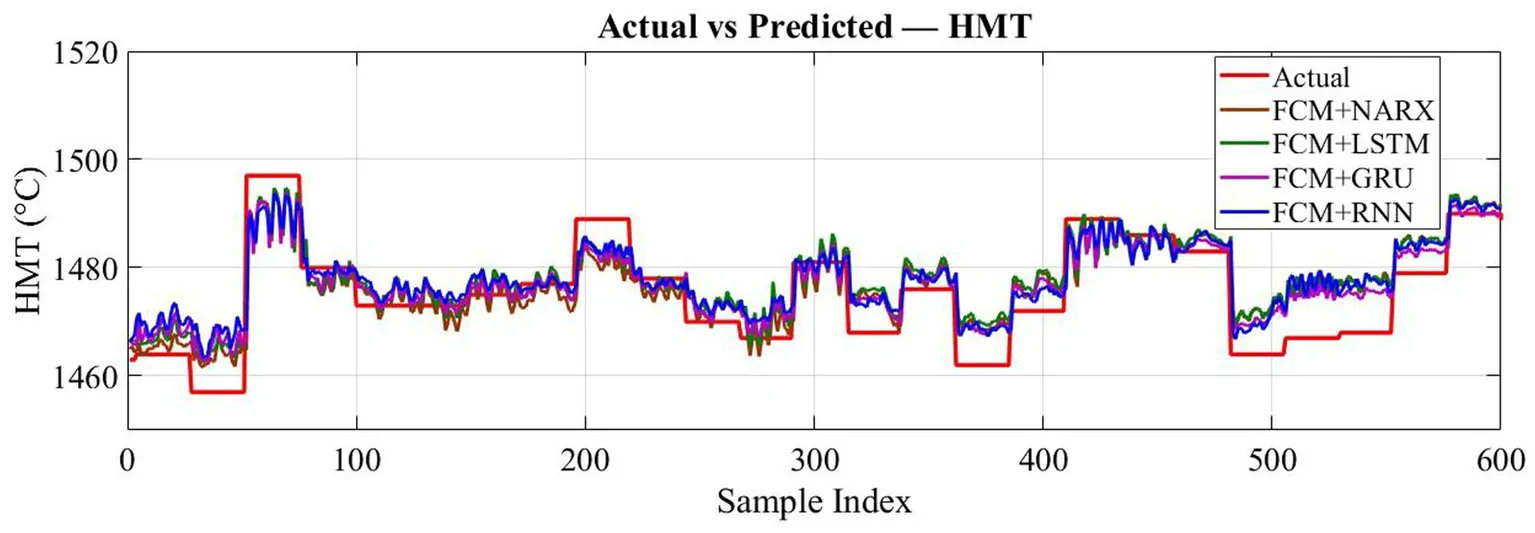
Actual vs. predicted of HMT.

**Figure 11:**
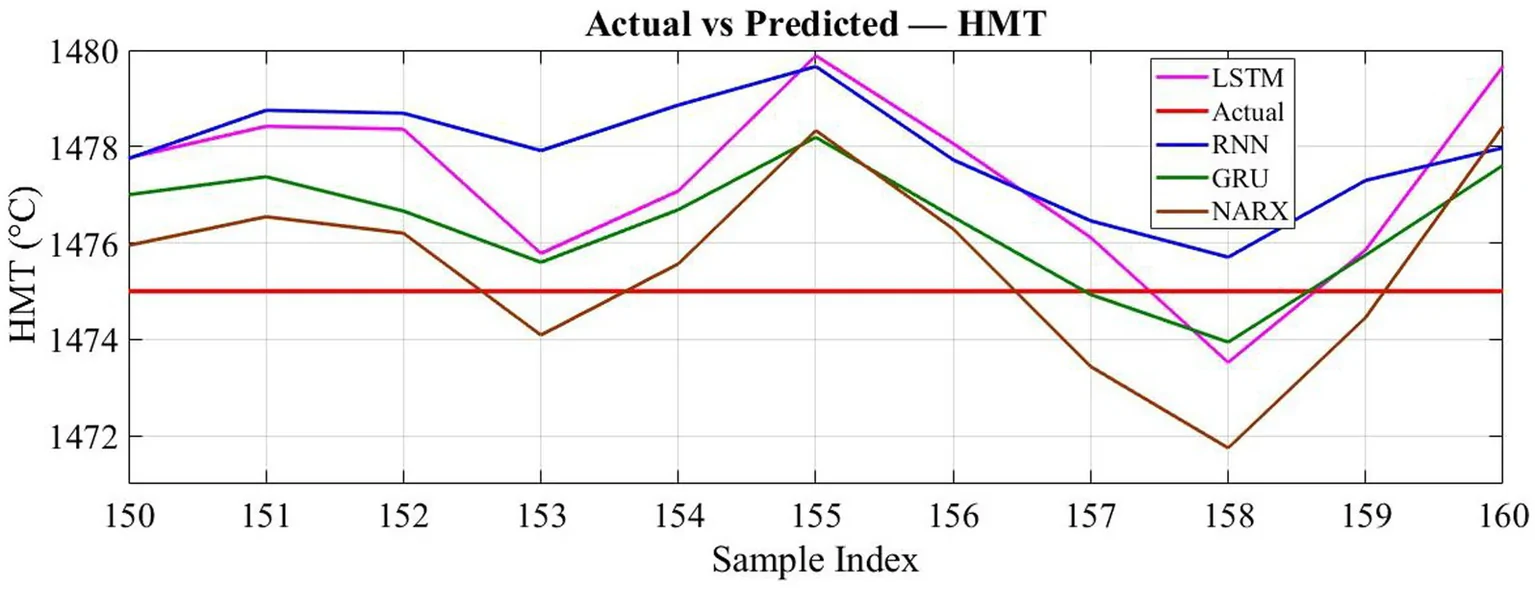
Zoom view of actual vs. predicted of HMT.

Figures 8–11 show the comparison of forecasted values to the measured data of HMT and CO₂% for four models. The results show that the GRU and LSTM models are highly accurate. Their prediction lines are fairly close to the actual data, and they are able to capture the sharp and stepwise changes in HMT and the rapid and high-frequency variations in CO_2_. The RNN and NARX models, on the contrary, performed poorly exhibiting the lag and smoothing effects. They also failed to react quickly to the sudden change in temperature in HMT. The two models had problems in replicating the real CO₂ data, which is a significant weakness of their model, replicating both sharp transients and oscillations.

**Figure 12:**
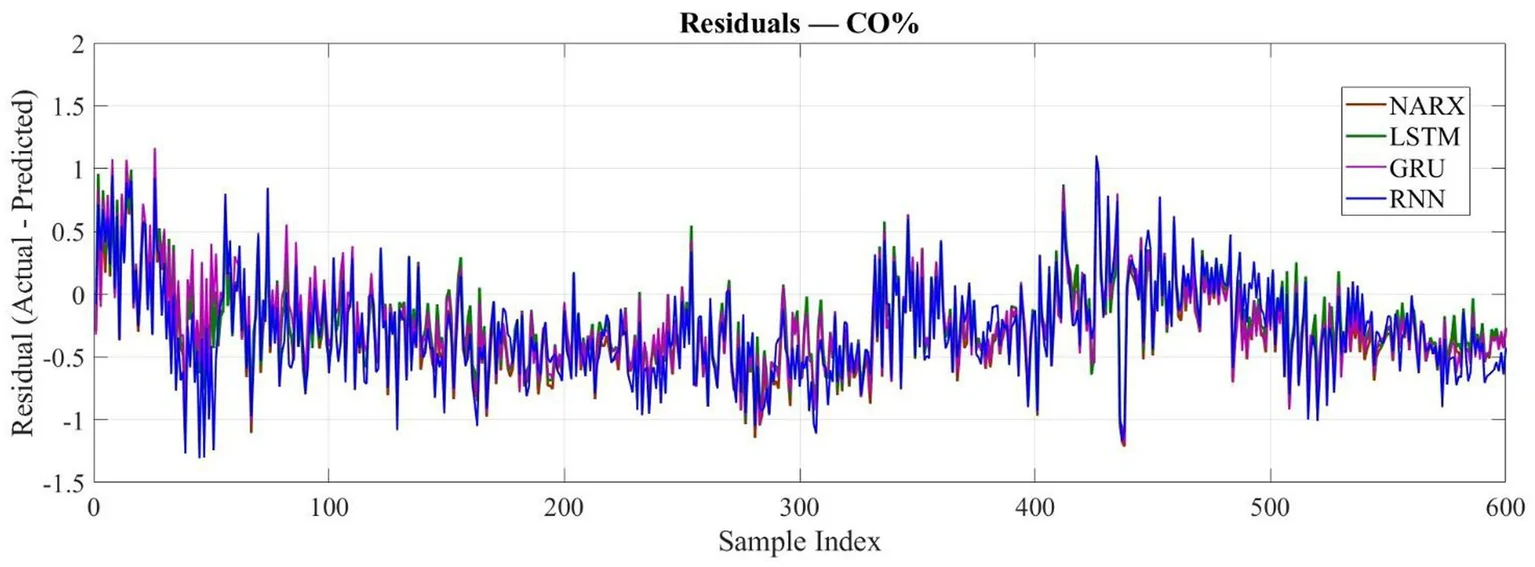
Residuals of CO %.

**Figure 13:**
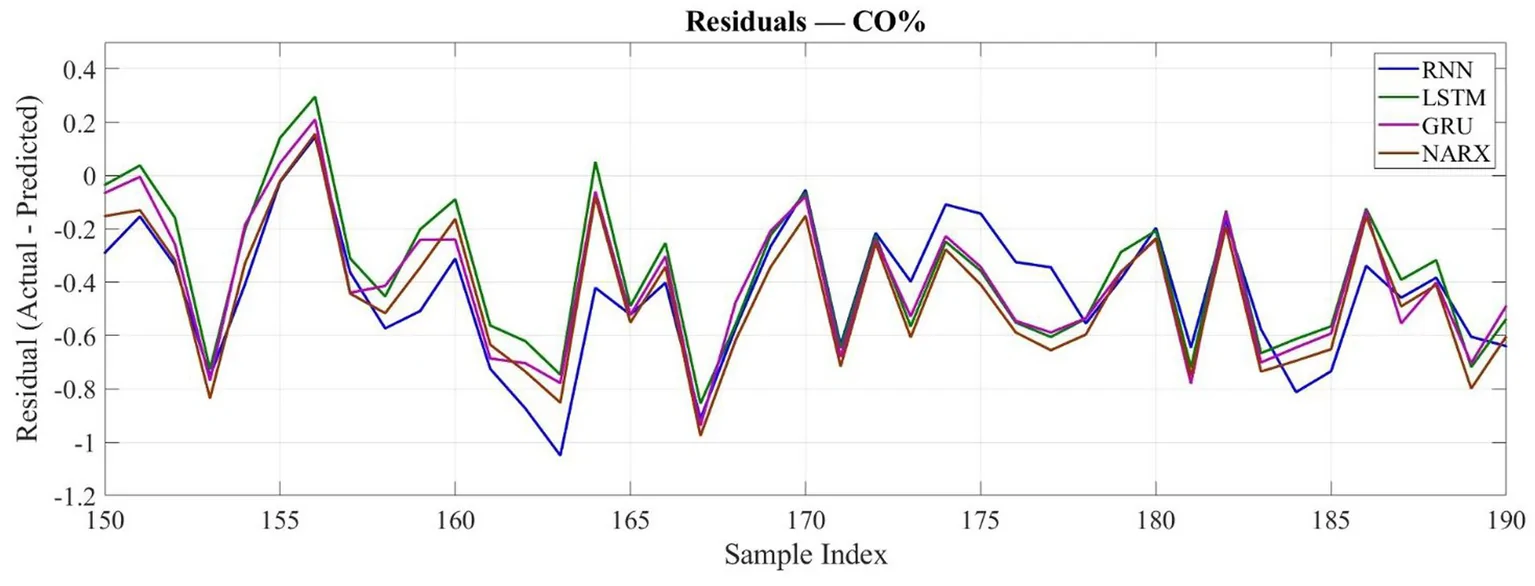
Zoom view of residuals of CO %.

**Figure 14:**
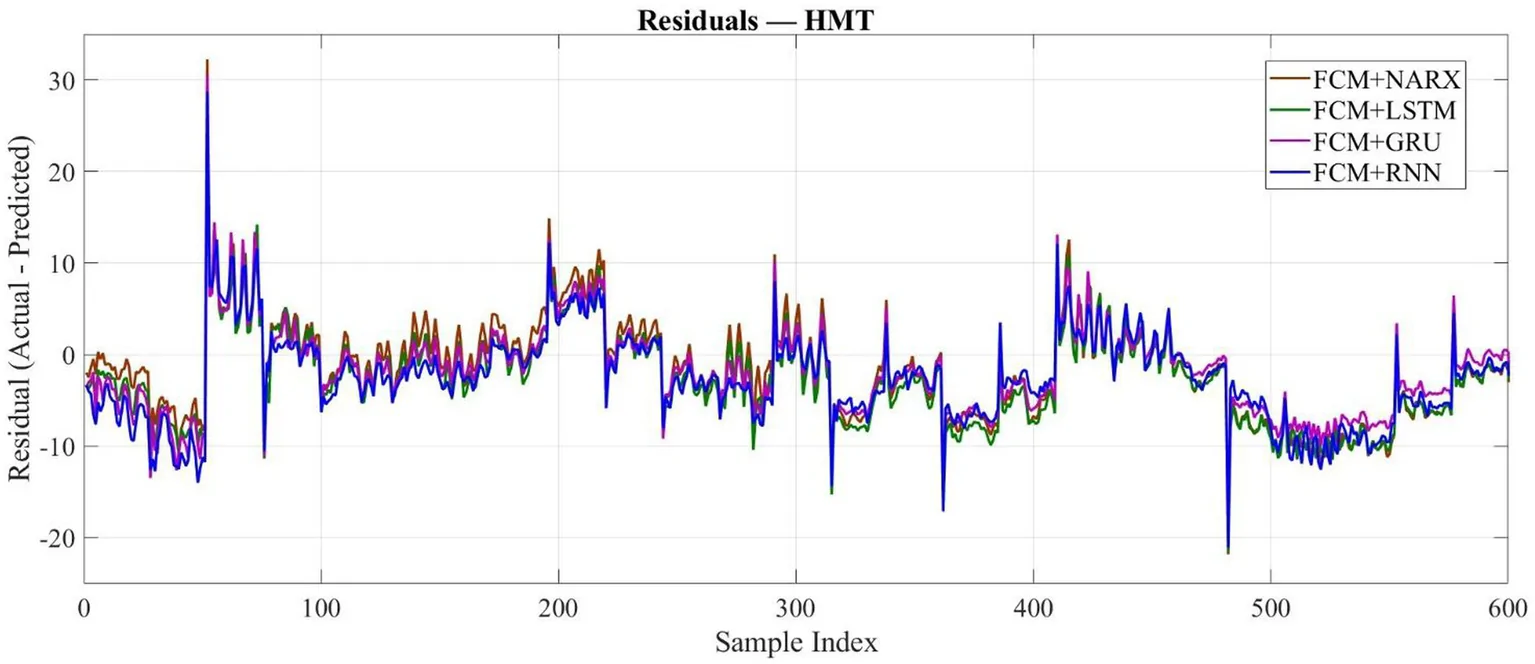
Residuals of HMT.

**Figure 15:**
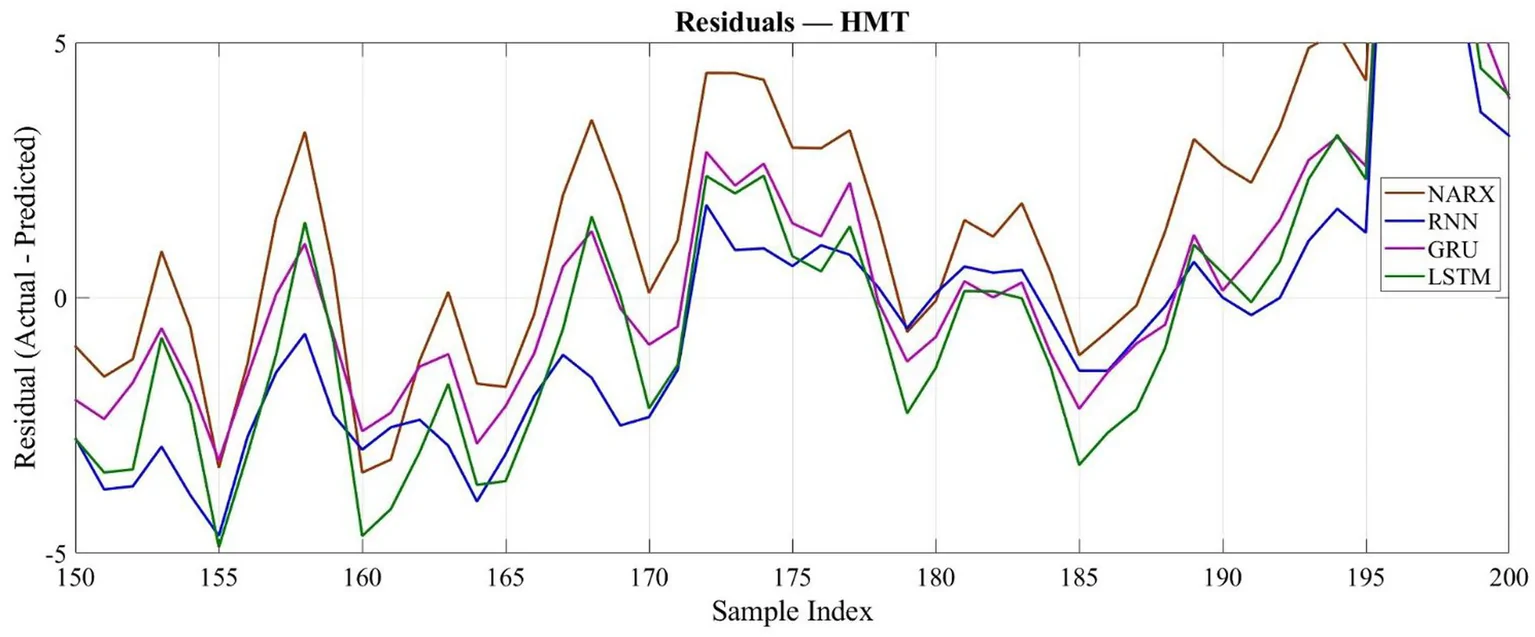
Zoom view of residuals of HMT.

Figures 12–15 indicate the time-series residuals that give a dynamic performance of the GRU, NARX, LSTM, and RNN models with FCM in predicting HMT and CO. The residual plots show that the two GRU models are excellent at representing the intricate time dynamics of the process to give consistent and reliable predictions on both variables. The LSTM, NARX, and RNN models failed, and unstable residual values were observed when there were significant shifts in the process, which led to unreliable predictions. These values demonstrate the dynamic error behavior, which is better in the case of GRU architectures to model the industrial process.

**Figure 16:**
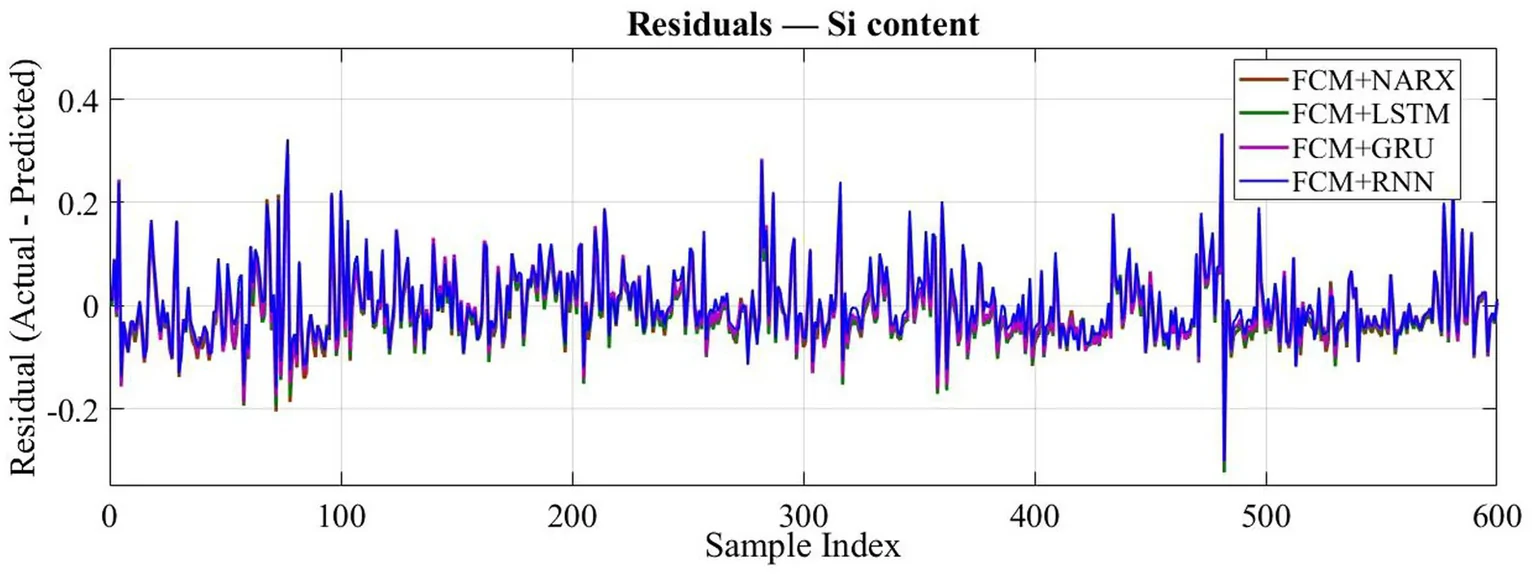
Residuals of Si.

**Figure 17:**
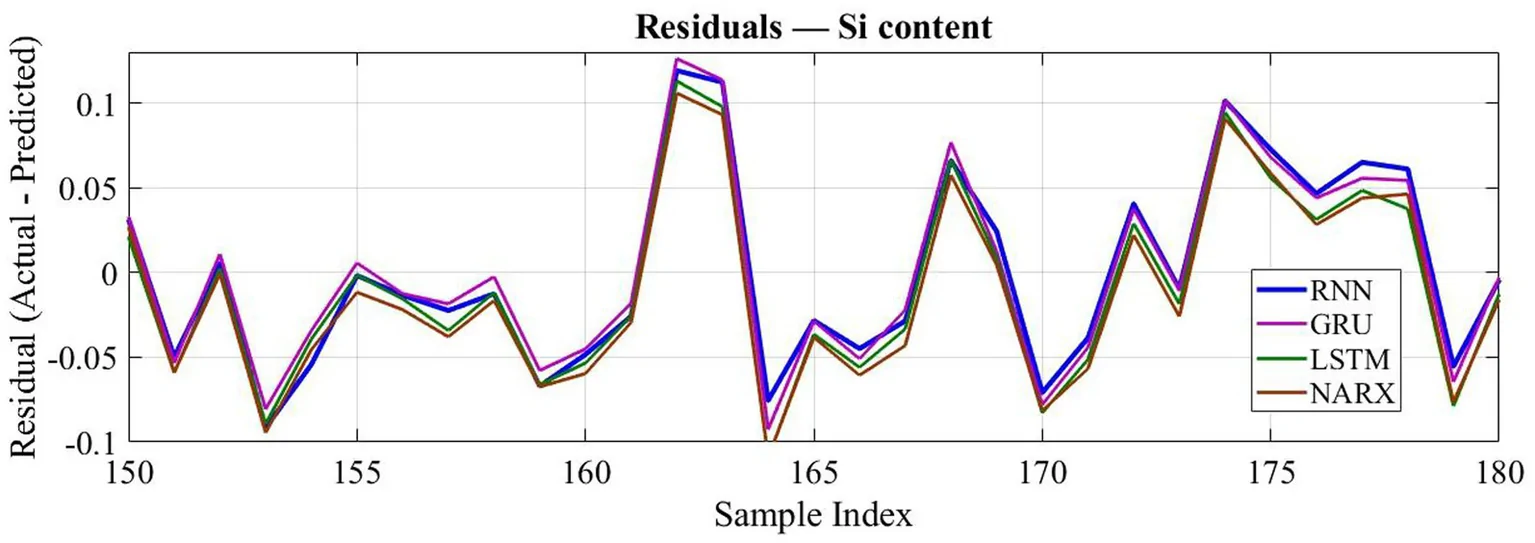
Zoom view of residuals of Si.

**Figure 18:**
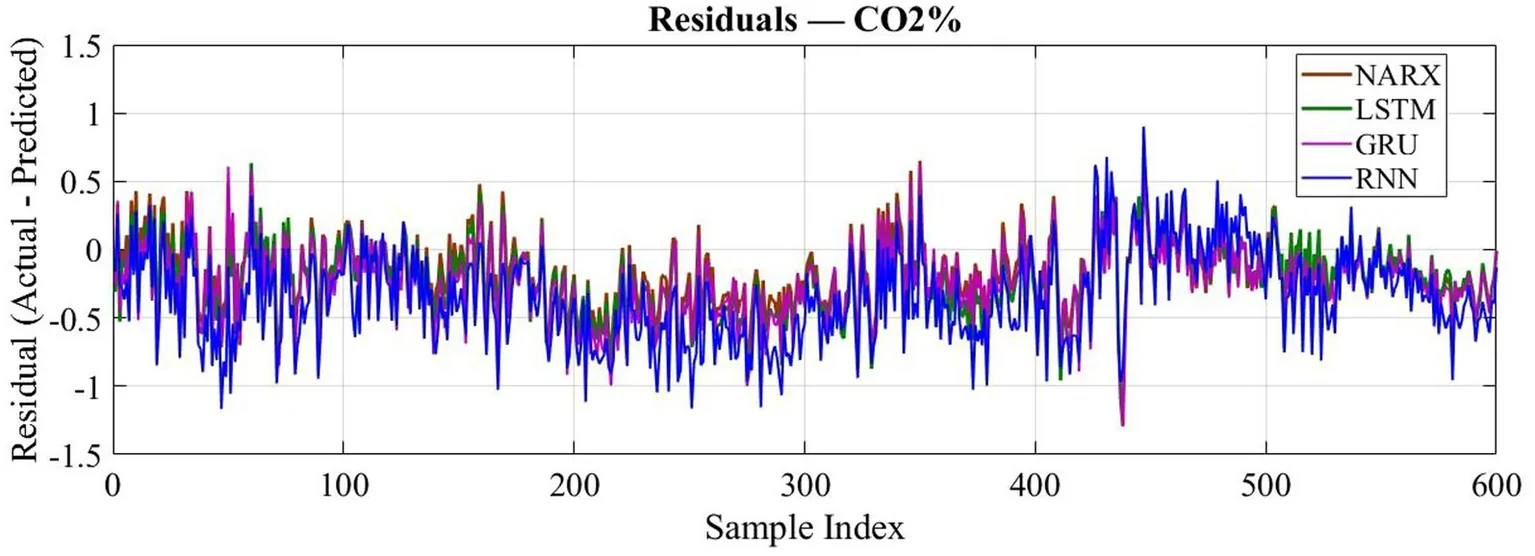
Residuals of CO_2_.

**Figure 19:**
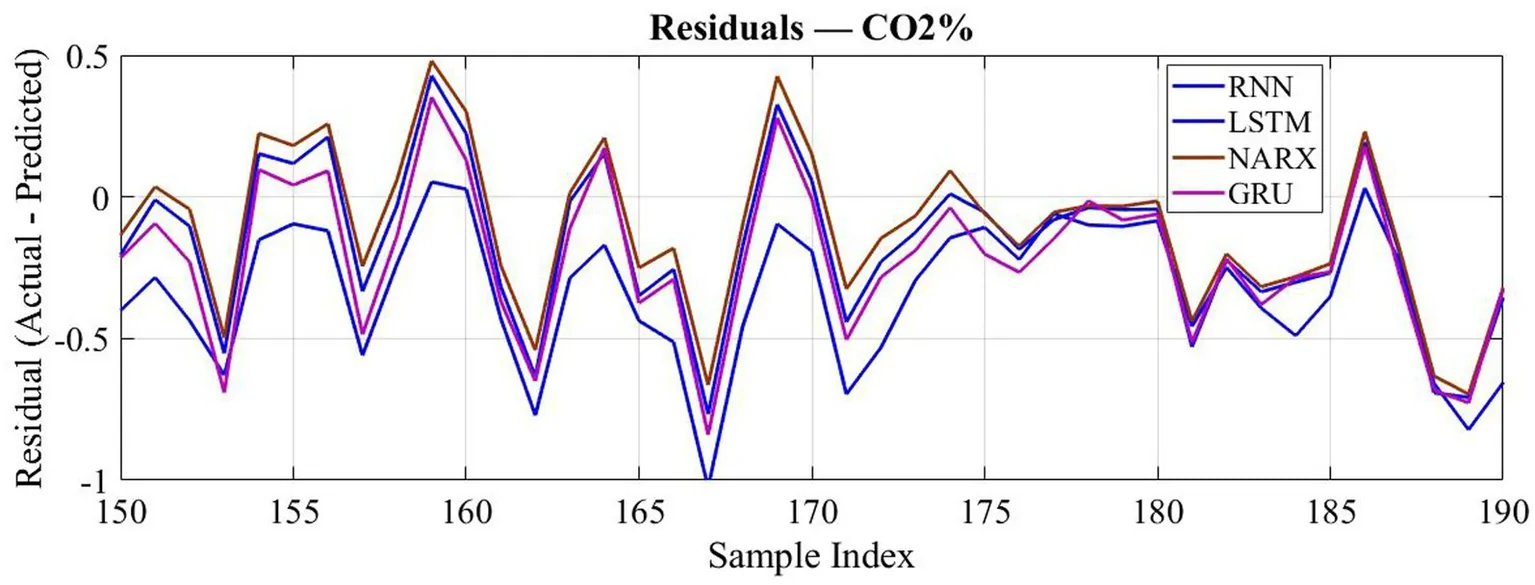
Zoom view of residuals of CO_2_.

Figures 16–19 indicate time-series residuals of predictions of Si content and CO₂, which represent the performance of the four hybrid models. The residual plots show that the LSTM and GRU models can capture the high-frequency variation of these process variables, and this can be evidenced by the fact that the error is low, meaning that the error is stable and predictive. On the other hand, the RNN and the NARX models recorded high prediction errors, with volatility. That means that their architectures could not cope with the dynamic nature of the blast furnace process and thus provided less predictive values. The results demonstrate that, the high tracking capacity of the GRU and the LSTM models, which proves their efficiency when it comes to predicting this variation of the complex process effectively.

### 5.1 Training and validation loss analysis

The loss function curves are preferred to evaluate the convergence behavior and learning performance of the developed hybrid models during training. The comparison of training and validation losses provides an indication of model stability and generalization capability.

**Figure 20:**
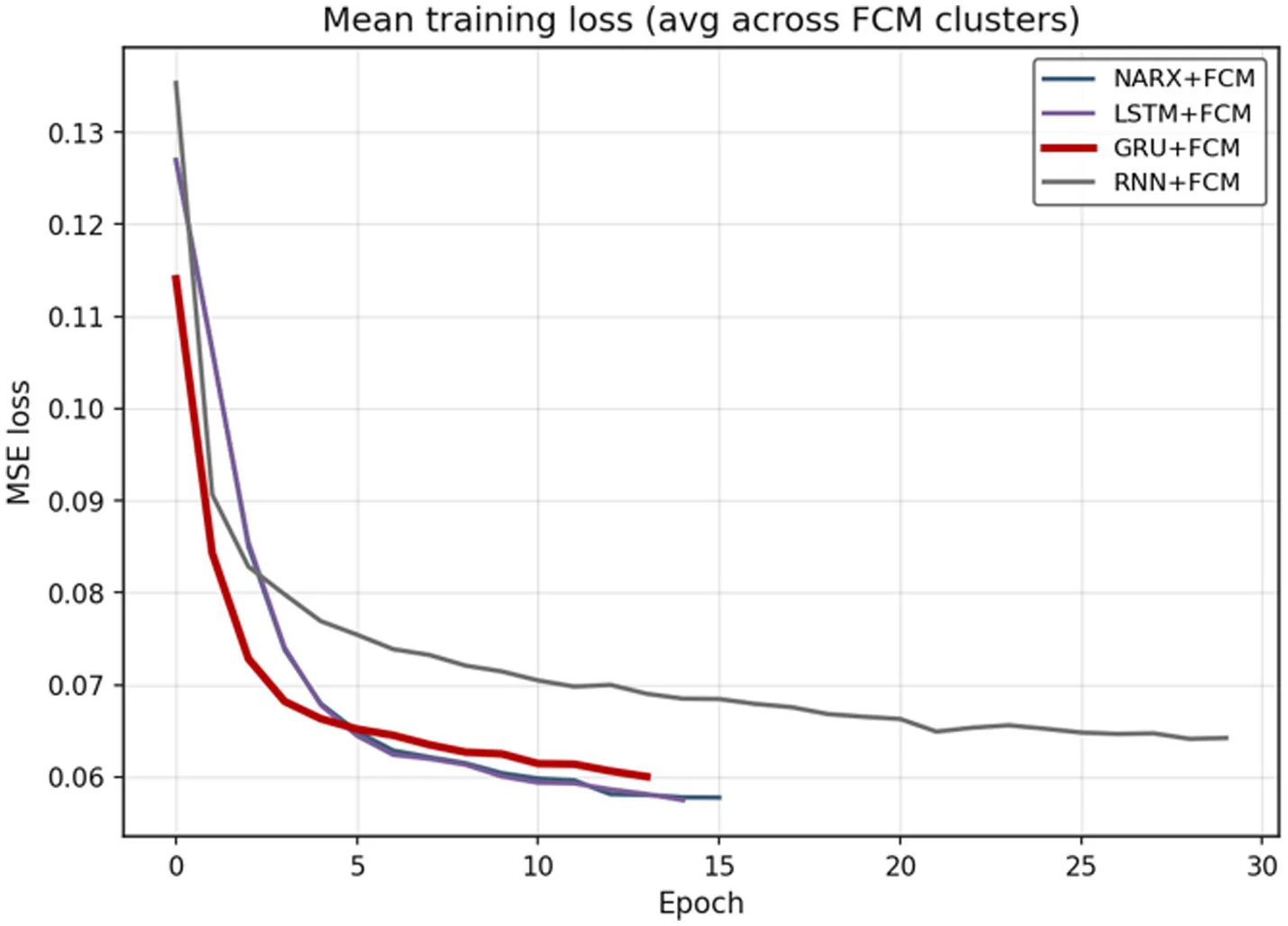
Training MSE loss curves.

**Figure 21:**
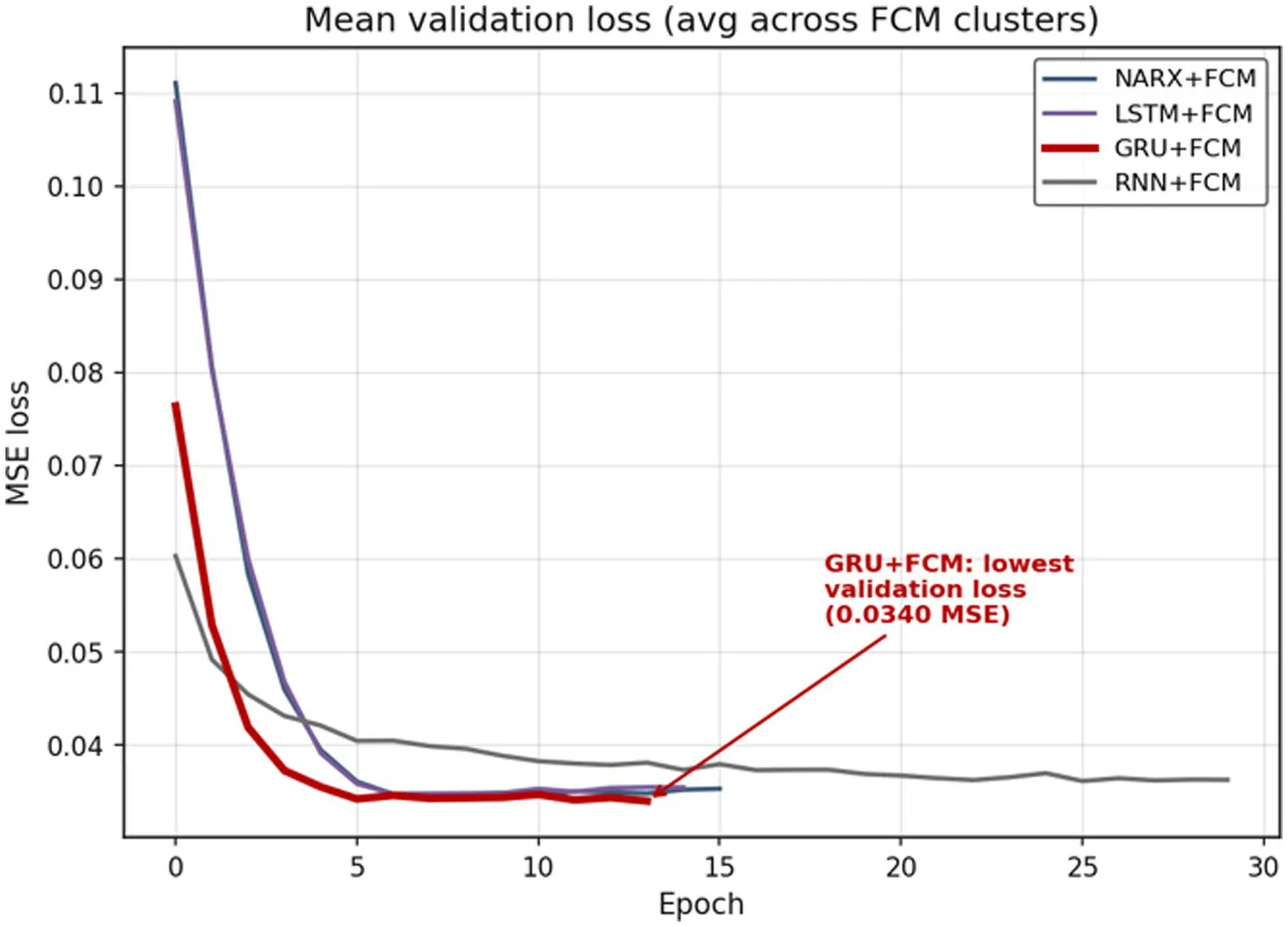
Validation MSE loss curves.

The MSE loss during training for the FCM with NARX, RNN, LSTM and GRU models are shown in Figure 20. For all models, there is a quick drop in training loss followed by consistent convergence. The model GRU + FCM is trained with the lowest training loss and the fastest convergence, indicating superior learning efficiency. The validation MSE loss curves for the FCM with DL models are illustrated in Figure 21. The validation loss is able to run down quickly and settle with little overfitting. The GRU + FCM model has got the lowest loss of 0.0348 MSE on validation set, which illustrates its best generalization performance and prediction ability.

### 5.2 Performance analysis from interpretation

The comparison of different DL algorithms, as shown in Table 4, which reveals that model performance depends on the target variable. Table 5 summarizes the performance of the different models and their computational complexity. The RNN model has the highest *R*^2^ value of 0.728 when predicting CO, whereas the LSTM model is the best in predicting HMT with the *R*^2^ value of 0.676. The GRU model gave the best results when used in predicting CO₂ with the *R*^2^ value of 0.912.

**Table 4:** Comparison of parameters with DL algorithms.

DL algorithms	Parameter	MAE	MSE	RMSE	*R*^2^ value
NARX	HMT	4.903	37.541	6.127	0.576
	Si content	0.056	0.005	0.072	0.328
	CO	0.433	0.272	0.521	0.700
	CO₂	0.295	0.134	0.366	0.428
RNN	HMT	5.068	38.682	6.220	0.563
	Si content	0.055	0.005	0.071	0.349
	CO	0.405	0.246	0.496	0.728
	CO₂	0.326	0.161	0.401	0.314
LSTM	HMT	4.242	28.685	5.356	0.676
	Si content	0.053	0.005	0.071	0.354
	CO	0.418	0.258	0.508	0.715
	CO₂	0.332	0.167	0.408	0.287
GRU	HMT	4.764	35.254	5.938	0.602
	Si content	0.052	0.005	0.070	0.355
	CO	0.460	0.312	0.559	0.656
	CO₂	0.181	0.05	0.224	0.912

**Table 5:** Comparison of model training time and complexity.

Model	Training time (s)	Complexity	Suitable for real-time
NARX	~8.7	Very High [O(*n*·*p*·*h*^2^ + *c*·*n*)]	Not ideal
RNN	~4.7	Medium–High [O(*n*·*t*·*h*)]	Moderate
LSTM	~6.4	High [O(*n*·*t*·*h*^2^)]	Moderate
GRU	~5.2	High [O(*n*·*t*·*h*^2^)]	Moderate

*n*, training samples; *t*, time steps; *h*, hidden units; *p*, past input/output lags; *c*, clusters.

LSTM model exhibits exceptional accuracy and efficiency with the lowest RMSE across board and the value of 0.508. The RNN model was more effective at learning long-term temporal dependencies, which led to good accuracy with CO% prediction, with a value of 0.728. However, it took a longer time to train it because the gating of the model was complicated. The GRU model was outstanding because of its low computation costs, and it converged with minimum resources, which was offset by a value of 0.912 of CO₂ %.

Meanwhile, the NARX model also performed competitively with a value of 0.428, especially in estimating CO₂%, due to its effective handling of temporal lags. Hence, LSTM and RNN showed better predictive accuracy, while GRU can be used in situations demanding quick training and deployment with limited computational resources. Although RNN and LSTM architectures are theoretically advantageous for modelling long-term dependencies, the effective memory horizon of the blast furnace process in this dataset is relatively short due to frequent operational interventions and closed-loop control actions. Under such conditions, GRU architectures can outperform gated networks by avoiding excessive smoothing and over-regularization, thereby responding more effectively to rapid process fluctuations. Table 5 shows that the GRU model provides the best trade-off between forecasting accuracy and computational cost, achieving superior prediction performance with lower training time than LSTM and significantly lower complexity than NARX, making it the most suitable candidate for real-time blast furnace monitoring.

## 6 Discussion

The summary of previous studies such as parameters prediction, algorithms, errors, outcomes and their limitations is given in Table 6. While most current research has focused on forecasting a single blast furnace parameter, e.g., silicon content (Si), HMT, or CO%. There is still a significant gap in integrated multi-parameter predictive models for thermal process monitoring. In this study, several hybrid ML models like NARX, RNN, LSTM, and GRU are integrated into one multi-output dashboard for real-time forecasting of key thermal and process parameters. This method not only increases the reliability of prediction and operational understanding of the thermal performance of the furnace but also remedies some of the limitations in existing research, such as no multi-parameter prediction, limited explainability, and constraints in real time implementation. Unlike previous studies that mainly predict a single blast furnace parameter, the proposed framework enables simultaneous forecasting of multiple key process variables using FCM-assisted deep learning models validated on real industrial data.

**Table 6:** Comparison of proposed work with existing works.

References	Parameter prediction				Algorithms used	Accuracy/errors	Outcomes	Limitations
	Si	HMT	CO₂	CO				
Hao et al. (2025)	✓				CNN	RMSE not reported	Effective multiscale Si prediction	Single output only
Paixão et al. (2025)		✓			ML-based forecasting	*R*^2^ ≈ 0.85–0.88	Reliable HMT forecasting	No gas or Si integration
Bhardwaj et al. (2024)	✓	✓			Improved AOA-twin SVM regression	RMSE: not reported/*R*^2^ ≈ 0.90–0.93	Accurate Si & HMT forecasting using optimization-based SVM	Limited explainability; narrower feature scope
Hamidi and Fakhroleslam (2025)	✓			✓	Data-driven ML framework	RMSE: not reported/*R*^2^: not reported	Multi variable prediction framework for BF	Performance metrics not fully reported; scalability not validated
Waqas and Humphries (2024)		✓			Dual-channel multimodal data fusion	RMSE: ~15% reduction/*R*^2^ ≈ 0.85	Improved HMT prediction by fusing multimodal data	Focused only on HMT; no integration with emissions or Si
Ławryńczuk and Zarzycki (2025)			✓	✓	ANN	RMSE: not reported/*R*^2^: not reported	Effective ANN-based prediction for EAF endpoints	Different process (EAF); limited BF relevance
Serefoglu Cabuk et al. (2024)		✓			Digital twin with soft sensor	RMSE: not reported/*R*^2^ ≈ 0.95	Accurate BF temperature field estimation	Focus only on temperature; not multi-output
Shi C. -Y. et al. (2024)					Attention-based deep learning	*R*^2^ ≈ 0.92	Accurate heat load prediction with attention mechanism	Focused only on heat load; not generalized to multi-parameters
Zhang et al. (2024)	✓				Deep learning	94–96%	Reliable silicon prediction	Data-specific to studied BF; limited generalization
Malashin et al. (2024)			✓	✓	Multiscale spectral graph wavelet NN	RMSE: not reported/*R*^2^ ≈ 0.95–0.96	Accurate BF gas utilization prediction	Model complexity; weak interpretability
Proposed work	✓	✓	✓	✓	NARX, LSTM, GRU, RNN hybrid	MAE.MSE, RMSE and *R*^2^ reported, GRU: *R*^2^ = 0.912	Unified predictive dashboard; real-time Si, HMT, CO, CO₂ forecasting with clustering	Cross-furnace validation

The proposed hybrid FCM–DL framework more advances BF forecasting by integrating the identification of the operating regime and temporal DL in a unified architecture. The FCM module classifies the operational data into segments of furnace state and allows the forecasting models to accurately capture the changes in the process nonlinearity under the dynamic furnace operation. This hybrid learning strategy improves feature representation and enhances the robustness of the temporal models compared with traditional single-stage prediction approaches (Hao et al., 2025; Bhardwaj et al., 2024; Waqas and Humphries, 2024). The FCM + GRU model performed the most optimally when compared to the other evaluated models because it can efficiently capture long-term temporal dependencies, has faster convergence rate, and low computational complexity. The proposed framework is suitable for reliable real-time implementation in industries and is a practical tool for decision support in BF process monitoring and control for intelligent blast furnace system.

## 7 Conclusion

This study presents a real-time multi-output forecasting framework for key blast furnace thermal and gas parameters, including Si content, HMT, and CO and CO₂ gas composition percentages. The main contribution of this work is the integration of FCM-based operating-regime identification with deep learning models for simultaneous multi-parameter forecasting, enabling more reliable real-time thermal-state assessment than conventional single-parameter prediction approaches. The proposed predictive framework, developed using NARX, RNN, LSTM, and GRU models, achieved *R*^2^ values ranging from 0.355 to 0.912 across different target variables, demonstrating its capability to capture the complex dynamics of blast furnace operations. The results indicate that CO% and HMT are strongly associated with combustion efficiency and heat transfer dynamics and can be predicted with high accuracy. In contrast, the prediction of silicon content and CO₂% exhibits comparatively lower accuracy, reflecting the highly nonlinear, thermally coupled, and complex characteristics of BF processes. The limitation of this study is that the proposed framework is developed and validated using offline historical industrial DCS data, which may not fully capture real-time process variations. Also, the present work focuses on one-step prediction, without examining long-horizon forecasting performance. Future work will focus on integrating online adaptive learning and multi-step forecasting strategies to enhance model robustness and practical applicability in continuously changing BF environments. Also, the proposed work can be extended to incorporate additional thermal and operational variables along with advanced sensor fusion techniques to further enhance model performance. Moreover, the integration of advanced DL architectures, such as attention-based Recurrent Neural Networks and transformer models, may improve the capability to capture long-term temporal dependencies in furnace operations.

## Data Availability

The original contributions presented in the study are included in the article/supplementary material, further inquiries can be directed to the corresponding author.
